# Recent insights into structures and functions of C-type lectins in the immune system

**DOI:** 10.1016/j.sbi.2015.06.003

**Published:** 2015-10

**Authors:** Kurt Drickamer, Maureen E Taylor

**Affiliations:** Department of Life Sciences, Imperial College, London SW7 2AZ, United Kingdom

## Abstract

The majority of the C-type lectin-like domains in the human genome likely to bind sugars have been investigated structurally, although novel mechanisms of sugar binding are still being discovered. In the immune system, adhesion and endocytic receptors that bind endogenous mammalian glycans are often conserved, while pathogen-binding C-type lectins on cells of the innate immune system are more divergent. Lack of orthology between some human and mouse receptors, as well as overlapping specificities of many receptors and formation of receptor hetero-oligomers, can make it difficult to define the roles of individual receptors. There is good evidence that C-type lectins initiate signalling pathways in several different ways, but this function remains the least well understood from a mechanistic perspective.

## Highlights

• Sugar-binding C-type carbohydrate-recognition domains fall in five structural groups.
• Structures for many of these domains, covering all of the groups, have been obtained.
• Not all human C-type lectins have clear orthologues in other mammals such as mice.
• Different mechanisms by which C-type lectins initiate signalling remain to be defined.
• Hetero-oligomeric receptors add to the complexity of overlapping specificities.

**Current Opinion in Structural Biology** 2015, **34**:26–34

This review comes from a themed issue on **Carbohydrate–protein interactions and glycosylation**

Edited by **Ten E N Feizi** and **Robert S Haltiwanger**

For a complete overview see the Issue and the Editorial

Available online 7th July 2015

**http://dx.doi.org/10.1016/j.sbi.2015.06.003**

0959-440X/© 2015 The Authors. Published by Elsevier Ltd. This is an open access article under the CC BY license (http://creativecommons.org/licenses/by/4.0/).

## Introduction

Complex oligosaccharides on cell surfaces and glycoproteins in blood and other biological fluids often serve as recognition signals that are bound by specific glycan-binding receptors known as lectins [1]. The C-type lectins are the largest and most diverse of the lectin families found in animals. C-type lectins contain an independently folding, modular carbohydrate-recognition domain (CRD) that in most cases binds sugars by ligation to Ca^2+^, making the sugar-binding activity Ca^2+^-dependent [2]. Many receptors containing C-type CRDs are found in the immune system, with functions including cell adhesion, glycoprotein turnover or pathogen recognition based either on recognition of endogenous mammalian glycans or on binding to glycans on micro-organisms.

The ways that some C-type lectins function in the immune system have been well established. For example, the importance of the selectins in interactions between leukocytes and endothelia is fully supported by phenotypes of knock-out mice and symptoms of a human congenital disorder of glycosylation that prevents synthesis of the fucosylated ligands recognized by the selectins [3, 4]. Similarly, mannose-binding protein deficiency in humans shows that antibody-independent fixation of complement triggered by binding of serum mannose-binding protein to sugars on the surface of pathogens is an essential part of the innate immune response in young infants [5].

This review considers recent advances in our understanding of how C-type lectins in the immune system work, with an emphasis on summarizing the currently available structural information and defining the areas where functional and mechanistic information is still outstanding.

## Structures of C-type lectins: the current position

**Figure 1:**
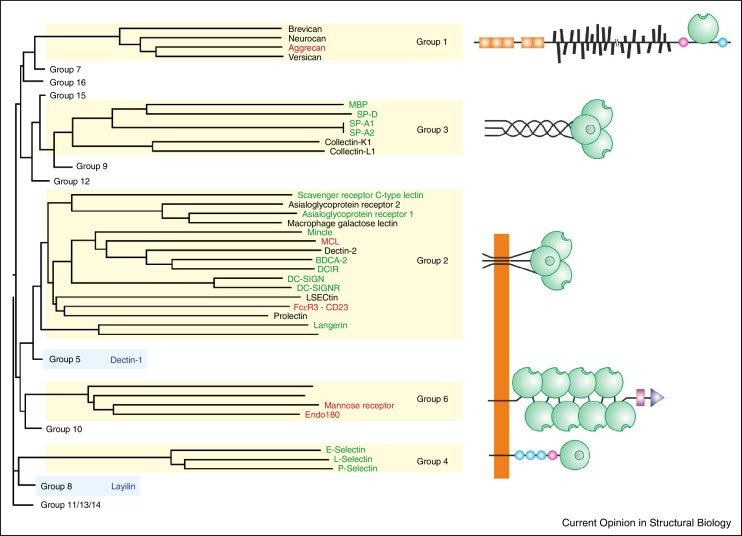
Summary of current state of structural analysis of glycan-binding C-type CRDs. Dendrogram at the left shows the relationships of the sequences of human CRDs, with the domains that bind glycans at the conserved Ca^2+^ site in yellow boxes and the domains that appear to bind glycans through non-canonical sites in blue boxes. Members of other groups that contain C-type lectin-like domains but lack key residues usually associated with Ca^2+^ and sugar-binding are not shown individually. Names of proteins containing the full set of residues needed to form canonical sugar-binding sites are shown in green when structures with bound glycan ligands have been obtained, in red for those cases in which unliganded structures have been determined and in black where structures have not been elucidated. The organization of proteins containing these CRDs is depicted schematically at the right, with the positions of CRDs, shown as green spheres, shown in relationship to other domains. Groups 2, 4 and 6 are receptors with transmembrane sequences. Proteins that bind sugars but lack the canonical binding site are indicated in blue. The crystal structure of dectin-1 has been determined and a possible mode of sugar binding has been suggested [63].

Structural analysis of the prototype C-type CRD from serum mannose-binding protein showed that residues conserved between C-type CRDs form a hydrophobic core and disulfide bonds that define the overall fold of the domain [6]. Additional conserved residues ligate a Ca^2+^ that forms the basis of a primary sugar-binding site. Many protein domains that have the C-type CRD fold lack the conserved Ca^2+^-binding site and thus do not bind sugars, which has led to the distinction between the sugar-binding C-type CRDs and the broader family of C-type lectin-like domains (CTLDs) [2]. About half of the CTLDs contain residues required for binding of the conserved Ca^2+^, and for the human proteins, Ca^2+^-dependent sugar binding has now been demonstrated for most of the domains that contain these residues. Crystal structures are available for the majority of these proteins, although not all are with bound glycan ligands (Figure 1). The CRDs that bind sugars through the canonical Ca^2+^-ligation mechanism fall in five of the structural groups defined by sequence similarity within the domain and the positions of the CRD in relationship to other protein domains (Genomics Resource for Animal Lectins; URL: http://www.imperial.ac.uk/research/animallectins). Structural information is available for members of each of these groups. Binding of sugars to two proteins that lack the canonical sugar-binding site, dectin-1 and layilin, suggests the presence of non-canonical sugar-binding sites in these proteins (Figure 1).

In all C-type CRD–glycan complexes examined so far, the presence of the principal Ca^2+^ results in ligation of a monosaccharide through two vicinal hydroxyl groups as originally seen for serum mannose-binding protein. Investigation of the full complement of human C-type lectins has largely borne out the early finding that the specificity for mannose-type sugars, which contain adjacent equatorial 3-hydroxyl and 4-hydroxyl groups (mannose, GlcNAc, and glucose) is determined by the presence of the residues EPN in the primary Ca^2+^ site while binding of galactose-type sugars in which the 4-hydroxyl group is axial (galactose and GalNAc) have QPD [7]. However, there have been some surprises. For example, the EPN-containing CRD of langerin, a pathogen-binding C-type lectin on Langerhans cells, binds mannose-type sugars at the primary-binding site but can also accommodate sulphated galactose at this site with the non-optimal Ca^2+^ ligation of galactose compensated by charge–charge interactions between the sulphate group and two lysine residues [8]. The ability of blood dendritic cell antigen 2 (BDCA-2) to bind galactose-terminated glycans in spite of the fact that it contains an EPN motif has recently been explained by demonstration that the mannose residue in terminal Galβ1–4GlcNAcβ1–2Man structures is in the primary binding site while galactose occupies an adjacent secondary site, making it somewhat analogous to mouse dendritic cell immunoreceptor 2 (DCIR2) [9••, 10••]. Finally, NMR analysis of ligand binding to DC-SIGN, a dendritic cell receptor that binds both high mannose oligosaccharides on viruses and Lewis^x^-containing glycans, suggests that in some cases crystallography may reveal only one of multiple modes of ligand binding [11, 12]. Multiple orientations of GalNAc-containing ligands bound to the macrophage galactose lectin have also been detected [13].

**Figure 2:**
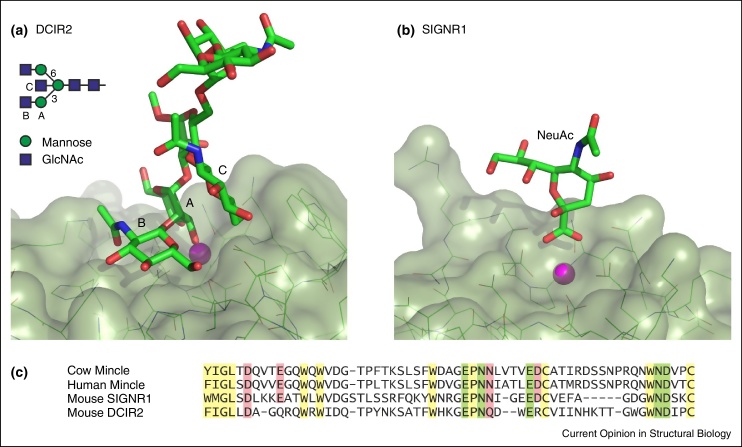
Recently reported structures of C-type CRDs with unusual modes of sugar binding. **(a)** Mouse dendritic cell immunoreceptor 2 (DCIR2) with a bound bisected biantennary N-linked glycan [PDB 3VYK. The positions of the three sugar residues A, B and C that interact with the protein are indicated in the schematic diagram of the bisected oligosaccharide. **(b)** Mouse SIGNR1 with bound sialic acid [PDB 4C9F]. In both (a) and (b), the primary Ca^2+^ bound to the protein is shown as a magenta sphere. **(c)** Sequence alignment of a portion of the CRDs from the proteins shown in (a) and (b) as well as mincle. Framework residues are highlighted in yellow, ligands for the conserved Ca^2+^ are highlighted in green and ligands for the adjacent accessory Ca^2+^ site in mincle are highlighted in pink.

In addition to the primary binding site, screening of glycan arrays and structural analysis of C-type CRDs in complex with glycan ligands has increasingly demonstrated the importance of contacts outside of the primary sugar-binding site in extended or secondary binding sites [14]. Examples include binding of high mannose oligosaccharides to DC-SIGN, binding of Lewis^x^-containing ligands to the scavenger receptor C-type lectin and the selectins, and binding of glycolipids such as trehalose dimycolate to the macrophage receptor mincle [15]. In addition to the examples described in the previous reviews [14, 15], extended site mechanisms for binding biantennary N-linked glycans have recently been demonstrated for the mouse dendritic cell immunoreceptor 2 (DCIR2) (Figure 2a) and BDCA-2 [9••, 10••].

At another extreme, a proposed binding interaction of sialylated glycans on IgG with SIGNR1, one of the mouse homologs of human DC-SIGN, is quite minimal: in the crystals, just the carboxyl group of the sialic acid interacts with the protein by making coordination bonds to the bound Ca^2+^ (Figure 2b) [16^••^]. However, a role for glycan recognition in binding of IgG to DC-SIGN has been difficult to document [17^••^]. While it may be that this controversy reflects difference between human DC-SIGN and mouse SIGNR1, it is also interesting to note that the position and ligation of Ca^2+^ in the crystals of mouse SIGNR1, obtained in the presence of high sulphate and low Ca^2+^, differs from that observed in most other CRD-sugar complexes and no adjacent second Ca^2+^ near the sugar-binding site is observed. This latter observation may be significant, because the four amino acid side chains that form this second Ca^2+^ site in human DC-SIGN and many other C-type CRDs are all present in SIGNR1 (Figure 2c). If the CRD in the structure of mouse SIGNR1 is not fully ligated with Ca^2+^, the conformation in the crystal may not reflect the organization of the binding site under physiological conditions. At low Ca^2+^ concentration, crystals of the CRD from human and mouse mincle, a macrophage receptor that binds glycoconjugates on the surface of mycobacteria and fungi, similarly lack the second Ca^2+^, resulting in a re-arrangement of the primary Ca^2+^ site [18, 19, 20•]. However, bound sugar ligand is only observed under conditions of higher Ca^2+^, when the second Ca^2+^ is occupied and the primary site takes on its canonical geometry.

In proteins such as mouse DCIR2 and BDCA-2, which lack the accessory Ca^2+^, residues that would have ligated this Ca^2+^ are changed so that a basic amino acid side chain takes up the position of the missing Ca^2+^ [9••, 10••]. Sugar binding to CRDs with a single Ca^2+^ site shows first order dependence on Ca^2+^ concentration, while CRDs with multiple Ca^2+^ sites have second or third-order dependence [9••, 20•]. The presence of additional sites also shifts the midpoint of the transition between sugar-binding and inactive conformations. The precise dependence on Ca^2+^ may be important in determining how sensitive CRDs are to variation in the Ca^2+^ concentration in various intracellular compartments, such as the endoplasmic reticulum, Golgi, and endosomes as well as regions of the extracellular matrix. In some cases, a sharp transition between active and inactive conformations, resulting from the higher order Ca^2+^ dependence, may be important for their biological functions, for example in ligand release from endocytic receptors in endosomes.

Knowledge of the mechanism of carbohydrate-recognition by C-type CRDs is now sufficient that glycomimetic drugs can be envisaged [21]. Mimics of sialyl-Lewis^x^ that inhibit binding of all three selectins have been developed through a fragment-based screening approach [22^••^]. One of these is now in clinical trials for sickle cell disease, where selectin-mediated interactions of leukocytes and platelets with vascular endothelium contribute to vasculo-occlusive crisis [23^••^].

## Relationships of C-type lectins across species

**Figure 3:**
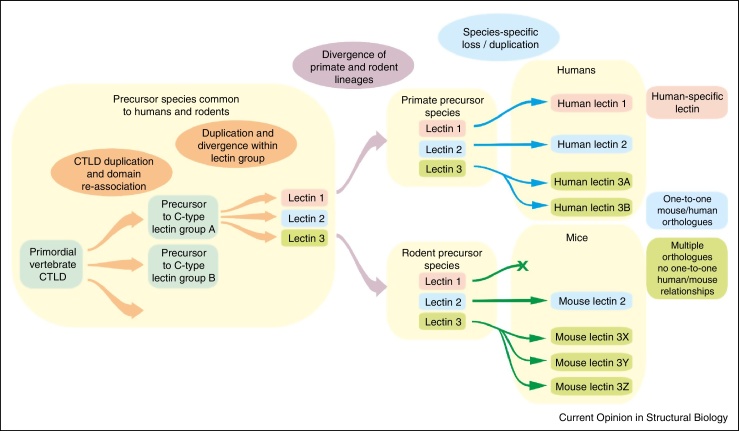
Summary of evolution of vertebrate C-type lectin-like domains. Common domain organizations were established early. However, recent evolution makes it difficult to define specific orthologues for some proteins, even between mammals such as humans and mice.

Comparison of C-type lectins in the immune systems of humans and mice shows that there are several distinct patterns of evolution (Figure 3). Conservation is observed for many receptors that bind endogenous glycans and function in adhesion and glycoprotein clearance by endocytosis. Examples of proteins for which it is possible to identify well-defined one-to-one orthologs between species include the selectin cell adhesion molecules [24] as well as the mannose receptor and the scavenger receptor C-type lectin, which function in clearance of serum glycoproteins released at sites of infection or inflammation [25, 26].

In contrast, many receptors that bind to pathogen glycans have undergone recent dramatic evolutionary changes, resulting in the absence of simple orthology between mice and humans. The differences include absence of specific proteins in one of the species and very recent duplications and divergences. For example, the human genome encodes two closely related proteins DC-SIGN and DC-SIGNR (L-SIGN) while there are eight genes for mouse SIGN proteins, none of which is organized in the same way as DC-SIGN and DC-SIGNR with extended neck domains between the CRD and the membrane [27, 28]. Similarly, in the collectin family, mice express two different forms of mannose-binding protein, while there is only one functional gene in humans [29]. It appears that there has been significant evolutionary pressure on some of these receptors during the recent evolution of mammals, possibly reflecting the fact that these receptors are targets for viruses that may have selected for changes in or loss of these genes. However, it is not an absolute rule that only receptors that bind endogenous ligands are conserved, since langerin and mincle both bind pathogen glycans but their properties are very similar across mammalian species [30, 31].

The absence of orthology between mice and humans for some proteins puts restrictions on the use of mouse models in understanding the functions of C-type lectins. In some cases, such as for the selectins and the mannose receptor, mice in which a particular gene is knocked out will provide information about the function of a well-defined human orthologue, while in other cases, including DC-SIGN and the mouse SIGNs, care must be taken to select an appropriate mouse model [28].

## Functions of C-type lectins in signalling

**Figure 4:**
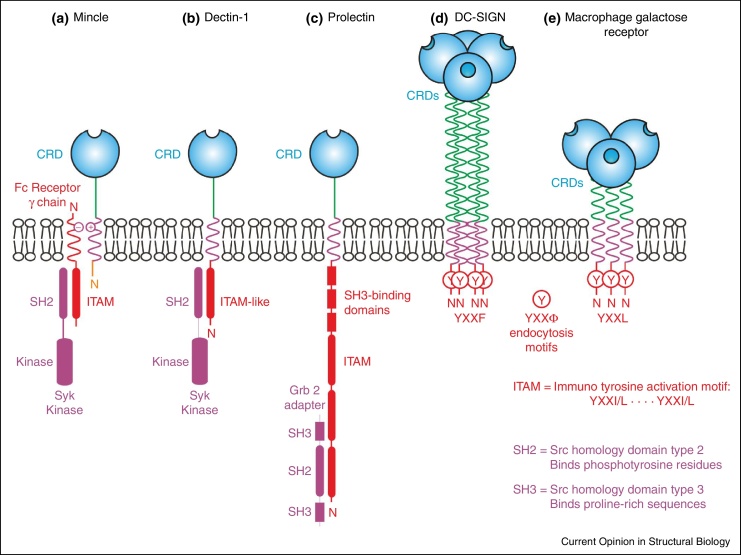
Organization of proteins containing extracellular C-type CRDs and intracellular domains involved in signalling. Sequence motifs in the cytoplasmic domains include immunotyrosine activation motifs (ITAMs), in which the tyrosine residues become phosphorylated, making them targets for binding to Src homology type 2 domains (SH2).

Many C-type lectins found on cells in the immune system have been reported to initiate intracellular signalling, but this remains the least well understood function of these receptors. The different arrangements of these receptors in the plasma membrane reflects the fact that there are several distinct mechanisms by which sugar binding at the cell surface leads to events on the cytoplasmic side of the membrane (Figure 4).

The cytoplasmic domains of two receptors, dectin-1 on macrophages and dendritic cells and prolectin on B cells, contain signalling motifs that allow direct activation of Syk kinase. Responses stimulated via dectin-1 signalling include phagocytosis, the respiratory burst and production of inflammatory cytokines such as TNF-α and IL-6 [32]. Dectin-1 appears to be important for anti-fungal immunity particularly against *Candida* in both humans and mice. Signalling through dectin-1 requires clustering and can be induced by dectin-1 binding to β-glucans on fungi including C*andida*, *Aspergillus* and *Pneumocystis* species [33]. Clustering leads to activation of the widely expressed protein kinase Syk via the sequence that resembles an immunoreceptor tyrosine-based activation motif (ITAM) in the cytoplasmic region of dectin-1. In many signalling pathways, tyrosine residues in ITAMs can be phosphorylated by Syk, which creates a Syk binding site that in turn results in the kinase becoming active on additional substrates. The particular targets vary depending on which ones are expressed in a particular cell type. In the case of dectin-1, downstream signalling events involve the CARD9/MALT1/Bcl-10 adapter complex. The consequences of ligand binding to prolectin are less well understood. In this instance, the cytoplasmic domain interacts with Grb2, an adapter protein that often recruits additional signalling molecules [34], but such binding partners have not yet been reported The presence of signalling motifs in the cytoplasmic domain of these sugar-binding receptors is reminiscent of the arrangement of another group of glycan-binding receptors, the siglecs [35].

Several other C-type lectins of myeloid cells, including mincle, dectin-2 and BDCA-2, lack intrinsic signalling domains but activate Syk through association with the common Fc receptor γ chain (FcRγ), which contains an ITAM. Signalling through association of FcRγ with mincle and dectin-2 occurs via activation of the CARD9/MALT1/Bcl-10 adapter complex by Syk, promoting outcomes that include secretion of cytokines TNF-α and IL-6 [36, 37, 38]. Antibodies to BDCA-2, which is a receptor of plasmacytoid dendritic cells, initiate signalling through the FcRγ chain, but in this case the result is suppression of inflammatory cytokines through a pathway involving BTK kinase, BLNK adapter protein and phospholipase Cγ2 [39]. BDCA-2 is found in primates and some more distantly related species, but the gene is not present in mice. The molecular mechanisms for stimulation of Syk, either directly by dectin-1 or indirectly through FcRγ, have not been determined. Clustering or crosslinking of receptors by ligand binding is likely to be important, but so far there is little information about the oligomeric state of mincle, dectin-2 or BDCA-2 or the stoichiometry of the complexes that form with FcRγ.

DC-SIGN and the macrophage galactose receptor (MGL) are both reported to modulate signalling pathways activated by toll-like receptors. It is proposed that the adapter LSP1 interacts with the cytoplasmic domain of DC-SIGN and becomes phosphorylated through a pathway initiated by toll-like receptor 4 [40]. Previous work showed that mannose-containing ligands, such as those found on *Mycobacterium tuberculosis*, lead to assembly of adapter proteins KSR1 and CNK and kinase Raf-1, enhancing proinflammatory cytokine production. Recent work now demonstrates that fucose-containing ligands, such as those present on *Helicobacter pylori*, result in binding of a different set of proteins, IKKɛ kinase and the deubiquitinating enzyme CYLD, leading to nuclear accumulation of kinase Bcl3 and suppression of the proinflammatory response [41]. From a mechanistic point of view, the basis for differential activation of DC-SIGN by different glycan ligands is difficult to understand, since structures of the unliganded CRD and the CRD bound to both mannose-containing and fucose-containing ligands, obtained both by crystallography [42, 43] and more recently by NMR [11, 12], do not indicate that there are changes in the protein structure upon binding of any of the ligands. In spite of evidence for activation of various downstream pathways by ligands for MGL, no specific binding partners have been proposed for MGL and less is known about the details of ligand binding [44]. The situation with these receptors may become clearer when the nature of the interaction between DC-SIGN and LSP1 has been defined. Both DC-SIGN and MGL mediate endocytosis [43, 45, 46] and have cytoplasmic domains containing YXXΦ motifs associated with trafficking through the clathrin-mediated endocytic pathway [47]. However, both receptors are pre-formed oligomers [48, 49] and in the case of DC-SIGN, the YXXL sequence resembles a hemi-ITAM, but does not function in this way [50].

## The importance of hetero-oligomers and spatial arrangements of CRDs

Binding of pathogen glycans by several C-type lectins, including DC-SIGN, MGL, serum mannose-binding protein and langerin is dependent on formation of homo-oligomers. Potential formation of hetero-oligomers creates an additional level of complexity when assessing the functions of C-type lectins in immunity against pathogens.

Recent evidence suggests that functions of both mincle and dectin-2 on macrophages are dependent on the macrophage protein MCL, a C-type lectin-like protein also known as dectin-3. The importance of mincle in recognition of the glycolipid trehalose dimycolate, a major virulence factor of mycobacteria and a potent stimulator of inflammatory responses, is indicated by complete abrogation of inflammatory response to trehalose dimycolate in macrophages deficient in mincle as well as in mincle knockout mice [51, 52]. However, MCL has also been shown to initiate signalling following stimulation with trehalose dimycolate [53^••^]. Formation of a functional complex of mincle with MCL and FcRγ has been suggested, based on co-immunoprecipitation of mincle and MCL when they are both expressed in 293T cells [54^••^]. But analysis of MCL-knockout mice suggests that MCL is required to up-regulate expression of mincle in response to stimulation with trehalose dimycolate and no evidence for hetero-oligomer formation was seen when MCL and mincle were co-expressed in the mouse macrophage-like RAW-264.7 cells [55^••^]. These findings support the alternative interpretation that initial sensing of trehalose dimycolate by constitutively expressed MCL stimulates signalling through activation of the CARD9/MALT1/Bcl-10 complex leading to activation of the transcription factor NFκB, which in turn up-regulates transcription of mincle mRNA. Binding of trehalose dimycolate to MCL has been described [19, 53••]. However, the C-type lectin-like domain of MCL does not contain the conserved residues needed for Ca^2+^-dependent sugar-binding of the trehalose moiety of trehalose dimycolate, or residues that form a hydrophobic groove seen in the mincle CRD that are predicted to accommodate the acyl chains of a glycolipid [18], so the mechanism of trehalose dimycolate binding to MCL is not clear.

The mannose-specific C-type lectin dectin-2 binds pathogens including mycobacteria and fungi such as *Candida* and *Malassezia*, which also interact with mincle but through different ligands: dectin-2 binds the capping mannose residues of lipoarabinomannan from mycobacteria or terminal mannose residues on fungal mannans [56•, 57•, 58•]. *Candida* mannan also binds weakly to MCL and MCL-knockout mice are more susceptible to *Candida* infection than wild type mice, suggesting a role for MCL in anti-fungal immunity, possibly through formation of a hetero-oligomer with dectin-2 [58^•^]. Dectin-2 has been seen to co-immunoprecipitate with MCL both in transfected RAW 264.7 cells and in mouse bone-marrow derived macrophages. In addition, bimolecular fluorescence complementation assays indicate formation of hetero-dimers between dectin-2 and MCL at the cell surface, although homo-dimers of dectin-2 and MCL also form [58^•^]. The relative importance of hetero-oligomers versus homo-oligomers and the exact nature of the interactions between the different receptors remain to be determined.

Structural analysis of oligomeric C-type lectins has shown the importance of the spatial arrangement of C-type CRDs in determining specificity for pathogen glycans. In one model, exemplified by serum mannose-binding protein, CRDs with low affinity and broad specificity are held in fixed geometrical arrangements in order to bind arrays of terminal sugars on bacterial and fungal cell surfaces [2]. In an alternative model, based on DC-SIGN, CRDs that bind restricted oligosaccharide motifs are flexibly linked and able to accommodate the disposition of these glycans on viral surfaces [59]. Results from recent quantitative imaging studies illustrate that, for cell surface receptors such as DC-SIGN, the arrangement of the oligomeric receptors on the cell-surface is also important for internalization of viruses [60^•^]. These methods reveal that DC-SIGN is clustered in microdomains on the plasma membranes of dendritic cells and on transfected cell lines and that each microdomain contains only 1–2 copies of the DC-SIGN tetramer. Internalization and infection studies with dengue virus, a small virus that binds to DC-SIGN, in cells transfected with DC-SIGN showed that no re-arrangement of micro-domains is required for efficient internalization of dengue virus and that the small number of DC-SIGN molecules in each micro-domain is sufficient to allow productive internalization and infection.

Spatial arrangement of the CRDs in the langerin trimer has also been studied in the context of formation of Birbeck granules, the specialized structures of the endosomal pathway with which langerin is associated in Langerhans cells [61]. Langerin expression is absolutely required for formation of Birbeck granules, as indicated by the absence of these structures in the langerin knock-out mouse and in humans with a very rare mutation in the langerin gene that prevents langerin expression [62]. Mutation of a residue located at the interface between adjacent CRDs in the langerin trimer has now been shown to cause destabilization of the langerin trimer and this mutation also causes abnormal membrane architecture in Birbeck granules as well as decreasing the affinity for HIV gp120 [61].

## Conclusions

The broad outlines of the mechanism by which simple sugars are bound to C-type CRDs, established roughly twenty years ago, remain valid. However, the ways that accessory binding sites lead to selectivity for specific classes of oligosaccharide ligands continue to be elucidated. While further structures of CRDs with bound ligand are needed, the current state of structural information makes it possible to envision that a relatively complete description of the sugar-recognition aspect of C-type lectin function can be achieved. The mechanisms by which these lectins participate in cell adhesion and in glycoprotein clearance are also now relatively well understood. In contrast, roles of C-type lectins in signalling are continuing to emerge and description of the mechanisms by which glycan binding leads to initiation of signalling pathways remains an area of active investigation.

## Conflict of interest statement

Nothing declared.

## References and recommended reading

• of special interest
•• of outstanding interest
